# Optimization of Acid Black 172 decolorization by electrocoagulation using response surface methodology

**DOI:** 10.1186/1735-2746-9-23

**Published:** 2012-12-11

**Authors:** Mahsa Taheri, Mohammad Reza Alavi Moghaddam, Mokhtar Arami

**Affiliations:** 1 Civil and Environmental Engineering Department, Amirkabir University of Technology, Tehran, Iran; 2 Associate Professor, Civil and Environmental Engineering Department, Amirkabir University of Technology, Tehran, Iran; 3 Textile Engineering Department, Amirkabir University of Technology, Tehran, Iran

**Keywords:** Acid Black 172, Decolorization, Electrocoagulation, Response surface methodology

## Abstract

This paper utilizes a statistical approach, the response surface optimization methodology, to determine the optimum conditions for the Acid Black 172 dye removal efficiency from aqueous solution by electrocoagulation. The experimental parameters investigated were initial pH: 4–10; initial dye concentration: 0–600 mg/L; applied current: 0.5-3.5 A and reaction time: 3–15 min. These parameters were changed at five levels according to the central composite design to evaluate their effects on decolorization through analysis of variance. High R^2^ value of 94.48% shows a high correlation between the experimental and predicted values and expresses that the second-order regression model is acceptable for Acid Black 172 dye removal efficiency. It was also found that some interactions and squares influenced the electrocoagulation performance as well as the selected parameters. Optimum dye removal efficiency of 90.4% was observed experimentally at initial pH of 7, initial dye concentration of 300 mg/L, applied current of 2 A and reaction time of 9.16 min, which is close to model predicted (90%) result.

## Introduction

Effluents from industries, such as textile, leather, plastics, paper, food and cosmetics contain many coloring substances, which can be toxic, carcinogenic and mutagenic [1-3]. In addition, some synthetic dyes cause allergy and skin irritation [4]. The dye-containing wastewater, are not only aesthetic pollutants, but also may prevent light penetration in water, and thereby damage water sources and ecosystem [5-7].

Electrocoagulation (EC) treatment process has been widely used due to its simplicity and efficiency [8-10]. In this process, generation of coagulants (iron or aluminum ions) by electrodissolution of the sacrificial anode(s) leads to formation of particles that entrap the pollutants [11-13]. The main reactions for dye removal using aluminum electrodes are as follows:

At the anode:

(1) $$\text{Al}\left(s\right)\to Al^{3+}+3e$$

At the cathode:

(2) $$3H_{2}O+3e\to \frac{3}{2}H_{2}\left(g\right)+3OH^{-}$$

In the solution:

(3) $$Al^{3+}+3H_{2}O\to \mathrm{Al}{\left(\text{OH}\right)}_{3}+3H^{+}$$

Response surface methodology (RSM) is a collection of mathematical and statistical techniques for modeling and analysis of problems in which a response of interest is influenced by set of independent variables [14,15]. Main advantages of optimization by RSM to conventional method are reduction of experimental trials in providing sufficient information for statistically valid results and evaluation of the relative significance of parameters and their interactions [16,17].

In recent years, the area of optimization dye removal efficiency by electrocoagulation has received enormous attentions [6,18-20]. However, according to our knowledge, application of RSM design in decolorization by EC rarely presented in scientific papers [21-24]. On the other hand, up to now there is no research available on treatment of diazo and metal-complex Acid Black 172 dye in aqueous media except by biological procedures.

The aim of the present study was to optimize Acid Black 172 dye removal from aqueous solution by electrocoagulation process using RSM. For this purpose, central composite design (CCD) was used to develop a mathematical correlation between Acid Black 172 dye removal efficiency and four selected independent parameters including initial pH, initial dye concentration, applied current and reaction time.

## Materials and methods

**Figure 1:**
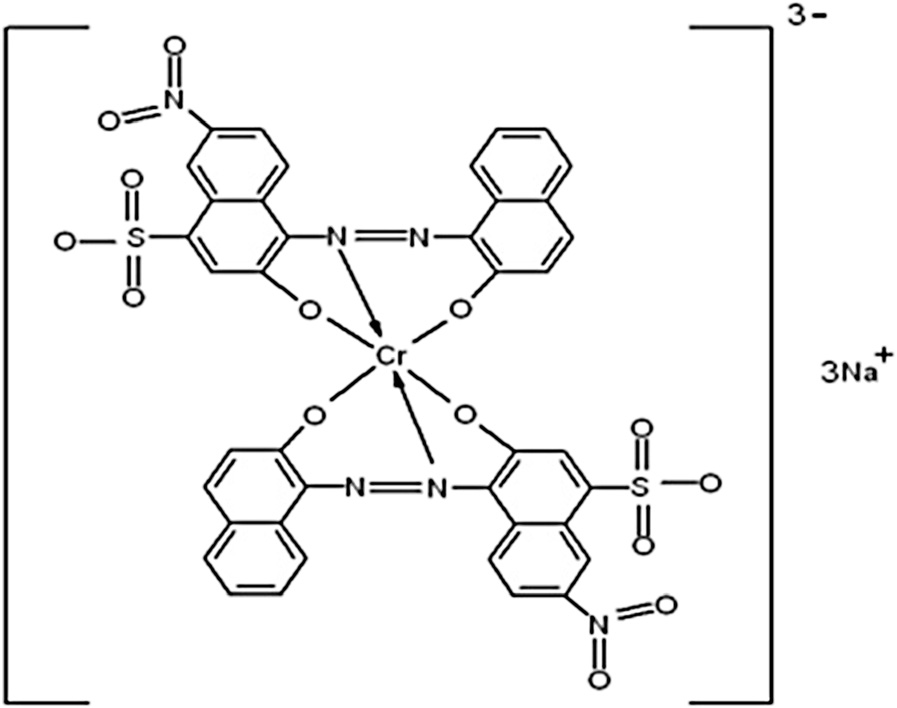
**The Chemical structure and characteristics of C.I. Acid Black 172 (molecular formula: C**_**40**_**H**_**20**_**CrN**_**6**_**Na**_**3**_**O**_**14**_**S**_**2**_**; λ**_**max**_**: 572 nm; molecular weight: 993.71 g**/**mol).**

Synthetic wastewater was prepared by dissolving Acid Black 172 which was provided by Alvan Sabet Company (Iran) in distilled water. The general properties and chemical structure of the selected dye is presented in Figure 1. A plexiglass cell with effective volume of 2.5 liters and four aluminum plates with total effective area of 240 cm^2^ were used; the thicknesses of aluminum plates were 3 mm and the distances between electrodes was kept constant at 3 cm. Electrodes were connected to a DC power supply (Micro, PW4053R, 0-5A, 0–40 V) in a monopolar mode. For preparing a mixed solution in EC cell, a magnetic stirrer (Velp, Scientifica, Italy) was used.

For preparation of stock solutions of the synthetic wastewater, Acid Black 172 dye as dissolved in deionized water and then diluted to obtain the desired concentrations. Sodium chloride (NaCl) was used to increase the conductivity of the solutions containing Acid Black 172 as the supporting electrolyte. The solution initial pH was adjusted before experiments by NaOH and H_2_SO_4_ and controlled using pH meter (340i, WTW, Germany). All the experiments were performed at room temperature. A total of 30 samples were taken from the cell at the end of experiments and centrifuged by a centrifuge device (Hettich, EBA 21, USA) at 5000 rpm for 5 min and then analyzed. Dye concentration was measured at a wavelength corresponding to the maximum absorbance (λ_max_) by UV-visible spectrophotometer (HACH, DR4000, USA).

For optimization of Acid Black 172 dye removal efficiency using CCD, 31 experiments consisting of 16 factorial points, 8 axial points (α = 2) and seven replicates at the center point were designed. Levels of selected parameters are shown in Table 1. As presented in Table 1, each independent variable was coded in 5 levels (−2, -1, 0, 1 and 2) as x_i_ according to Equation 4:

(4) $$x_{i}=\left(X_{i}-X_{0}\right)/\mathrm{\Delta X}$$

where X_0_ is value of the X_i_ (selected parameters) at the center point and ΔX presents the step change. Acid Black 172 removal efficiency was taken as the response of the experiments according Equation 5:

(5) $$Y_{i}=b_{0}+\sum_{i=1}^{n}b_{i}x_{i}+\sum_{i=1}^{n}b_{\mathrm{ii}}{x_{i}}^{2}+\sum_{i=1}^{n-1}\sum_{j=i+1}^{n}b_{\text{ij}}x_{i}x_{j}$$

where Y_i_ is the percentage of dye removal efficiency

**Table 1:** Experimental range and levels of independent parameters

**Parameters**		**Levels**				
		- **α**	−**1**	**0**	**1**	**α**
Initial pH	X_1_	4	5.5	7	8.5	10
Initial concentration (mg/L)	X_2_	0	150	300	450	600
Applied current (A)	X_3_	0.5	1.25	2	2.75	3.5
Reaction time (min)	X_4_	3	6	9	12	15

b_0=_ the constant coefficient

b_i_ = the regression coefficients for linear effects

b_ii_ = the quadratic coefficients

b_ij_ = the interaction coefficients

and x_i_, x_j_ are the coded values of the parameters.

The statistical software “Minitab”, version 15.1.1.0 was used for the regression and graphical analyses of the experimental data obtained. The accuracy of the fitted model was justified through analysis of variance (ANOVA) and the coefficient of R^2^.

## Results

### Development of regression model equation and validation of the model

The design matrix with experimental and predicted Acid Black 172 removal efficiencies are listed in Table 2. The final model is expressed by:

(6) $$\begin{array}{l} Y=89.5643-3.7133_{x1}-9.0542_{x2}+5.9642_{x3}\\ \;+8.1442_{x4}-0.5629_{x1^{2}}-0.5442_{x2^{2}}\\ \;-3.0404_{x3^{2}}-3.7967_{x4^{2}}+0.2138_{x1x2}\\ \;+0.385_{x1x3}+0.2713_{x1x4}+4.4125_{x2x3}\\ \;+5.3913_{x2x4}-1.3875_{x3x4} \end{array}$$

**Table 2:** RSM design and experimental and predicted values

**Run**	**Initial pH (x**_**1**_**)**	**Initial dye concentration (x**_**2**_**)**	**Applied current (x**_**3**_**)**	**Reaction time (x**_**4**_**)**	**Dye removal (%)**	
					**Experimental**	**Predicted**
1	−2	0	0	0	96.64	94.74
2	0	0	−2	0	58.89	65.47
3	−1	−1	−1	−1	93.01	89.57
4	0	0	0	0	89.33	89.56
5	0	0	0	0	89.91	89.56
6	0	0	2	0	91.33	89.33
7	0	2	0	0	70.19	69.28
8	1	1	−1	−1	43.7	43.11
9	1	−1	1	−1	87.16	87.05
10	−1	−1	−1	1	96.42	97.3
11	0	0	0	−2	50.43	58.09
12	0	0	0	0	89.99	89.56
13	−1	−1	1	−1	95.31	94.67
14	1	−1	−1	1	89.78	89.22
15	−1	−1	1	1	97.26	96.86
16	1	1	−1	1	76.46	73.5
17	−1	1	−1	1	81.6	80.73
18	0	0	0	0	90.44	89.56
19	0	0	0	0	89.85	89.56
20	1	−1	−1	−1	89.36	80.4
21	0	0	0	2	93.74	90.67
22	1	1	1	−1	71.89	67.41
23	2	0	0	0	73.4	79.89
24	1	1	1	1	89.79	92.25
25	0	0	0	0	88.65	89.56
26	−1	1	1	1	92.57	97.93
27	1	−1	1	1	92.67	90.32
28	0	0	0	0	88.78	89.56
29	−1	1	1	−1	74.61	74.18
30	−1	1	−1	−1	52.67	51.42
31	0	−2	0	0	100	105.5

**Figure 2:**
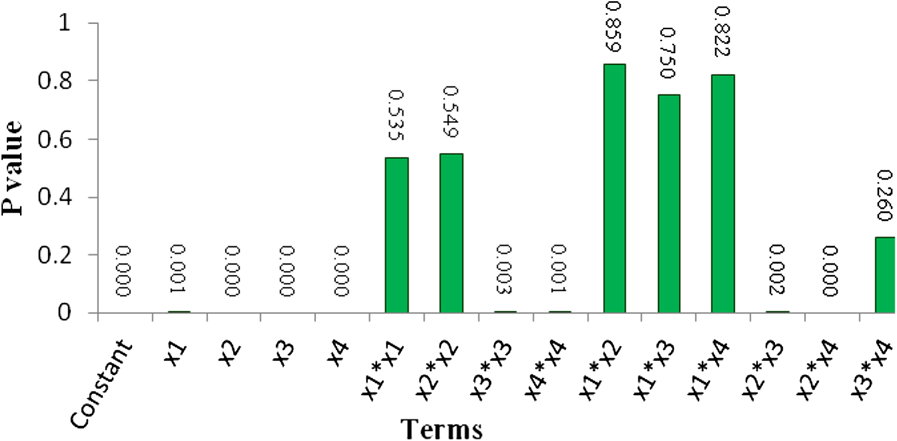
Estimated P values of the parameters for Acid Black 172 removal efficiency (%).

**Figure 3:**
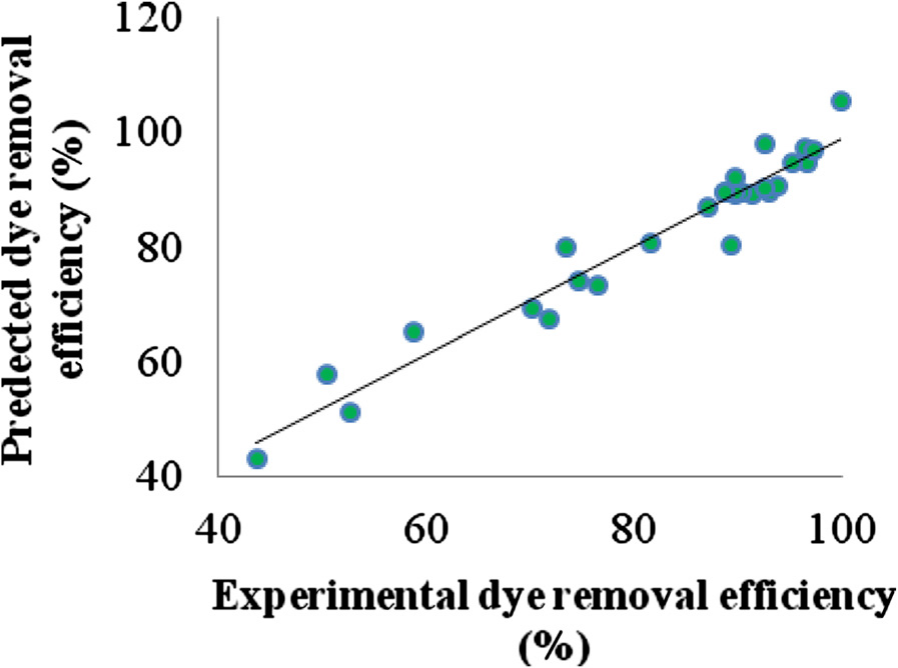
**Parity plot for the experimental and predicted value of Acid Black 172 removal efficiency (%).**

Estimated P values of the parameters for Acid Black 172 removal efficiency (%) are illustrated in Figure 2. As depicted in Figure 2, the amounts of P (P = 0.00) for all independent parameters confirms that four selected factors are significant. However, it was found that all square and interaction terms except x_1_ ^2^, x_2_ ^2^, x_1_x_2_, x_1_x_3_, x_1_x_4_ and x_3_x_4_ (P ≥ 0.05) were significant to the response. The analysis of variance (ANOVA) for the Acid Black 172 dye removal efficiency is given in Table 3, According to this table, the P value of 0 (P ≤ 0.05) justifies the reliability of the fitted polynomial model through ANOVA with 95% confidence level. Furthermore, parity plot for the experimental and predicted value of Acid Black 172 removal efficiency (%) is demonstrated in Figure 3. High R^2^ value of 94.48% validates the statistical significance of the model for the selected dye removal.

**Table 3:** **Analysis of variance** (**ANOVA**) **for Acid Black 172 removal efficiency** (%)

**Source**	**DF**	**Seq SS**	**Adj SS**	**Adj MS**	**F**	**P**
Regression	14	6169.47	6169.47	440.68	19.55	0
Linear	4	4743.97	4743.97	1185.99	52.61	0
Square	4	613.84	613.84	153.46	6.81	0.002
Interaction	6	811.65	811.65	135.28	6	0.002
Residual Error	16	360.7	360.7	22.54		
Lack-of-Fit	10	358.04	358.04	35.8	80.91	0
Pure Error	6	2.66	2.66	0.44		
Total	30	6530.17				

Note: R^2^ = 94.48%, R^2^ (adj) = 89.64%.

**Figure 4:**
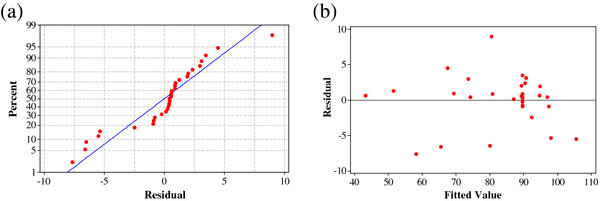
**(a) Normal probability plot and (b) residual versus fit plot for Acid Black 172 removal efficiency (%).**

In addition, normal probability and residuals versus fitted values plots for Acid Black 172 removal efficiency are illustrated in Figure 4. As seen in Figure 4(a), the normality assumption was relatively satisfied as the points in the plot form fairly straight line. The reliability of the model was also examined with the plot of residuals versus fits in Figure 4(b). As illustrated in this figure, no series of increasing or decreasing points, patterns such as increasing residuals with increasing fits and a predominance of positive or negative residuals should be found. As a result, Figure 4 shows that the model is adequate to describe Acid Black 172 removal efficiency by response surface methodology.

### Effects of operating parameters

**Figure 5:**
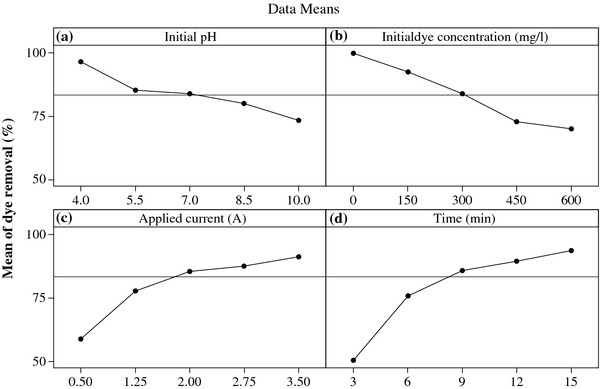
**Main effect plots of parameters for Acid Black 172 removal efficiency: (a) initial pH, (b) initial dye concentration, (c) applied current and (d) reaction time.**

**Figure 6:**
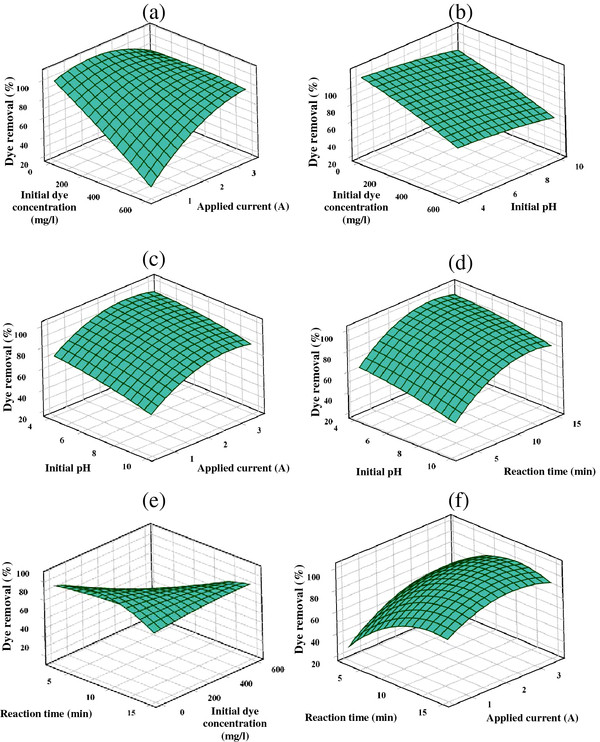
**Surface plots as a function of: (a) initial dye concentration and applied current; (b) initial dye concentration and initial pH; (c) initial pH and applied current (d) initial pH and reaction time; (e) reaction time and initial dye concentration; (f) reaction time and applied current.** Hold values: (initial pH =7, initial dye concentration =300 mg/L, applied current =2 A, and reaction time = 9 min).

The main effect of each parameter on the Acid Black 172 removal efficiency is shown in Figure 5. For a better explanation, 3D plots are also presented in Figure 6. As illustrated in Figure 5, by decreasing in initial pH and initial dye concentration, and increasing in applied current and reaction time, dye removal efficiency improved. For instance, Acid Black 172 removal efficiencies decreased from 96.6% to 73.4% with the increase in initial pH from 4 to 10, respectively. In this investigation, according to Figure 5(a), best performances of EC system for dye removal were obtained at initial pH of 4.

### Process optimization

In order to determine the optimum point by electrocoagulation process, the desired objective in terms of Acid Black 172 removal efficiency was defined as target to achieve 90% removal efficiency. Table 4 shows the optimum values for Acid Black 172 removal from aqueous solution. The first row of this table is optimal conditions without any starting value. The optimum points from second to fifth rows in Table 4 was obtained with consideration of 4, 0.5 A and 3 min, as starting values for initial pH, applied current and reaction time, respectively. The initial dye concentrations of 150, 300, 450 and 600 mg/L were selected for second, third, fourth and fifth rows as starting values, correspondingly. As reported in Table 4, the experimental dye removal efficiencies and RSM predictions are in close agreement.

**Table 4:** Optimum values for Acid Black 172 removal from aqueous solution

**No**	**Initial pH**	**Initial dye concentration (mg**/**L)**	**Applied current (A)**	**Reaction time (min)**	**Dye removal efficiency (%)**	
					**Predicted**	**Experimental**
1	7	300	2	9.16	90	90.4
2	4	150	1.76	4.37	90	91.96
3	4	300	2.78	6.72	90	94.26
4	4	450	3.3	8.41	90	95.2
5	4	600	3.5	9.1	90	94.57

### Dye removal kinetic

**Figure 7:**
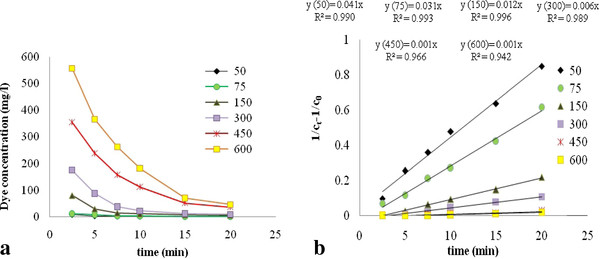
**(a) Variation of Acid Black 172 concentrations with time; (b) determination of the kinetic constants for Acid Black 172 removal (initial pH of 7 and applied current of 2 A).**

The influence of reaction time on dye removal at different initial concentrations is illustrated in Figure 7(a). Second order kinetic model according to Equation 7 is:

(7) $$1/C_{t}-1/C_{0}=\text{kt}$$

where C_t_, C_o_, and k are dye concentrations at any time t, initial dye concentration, and kinetic constant, respectively. Plots of (1/C_t_-1/C_0_) with time are shown in Figure 7(b) for various initial dye concentrations (from 50 to 600 mg/L), at initial pH of 7 and applied current of 2 A. As demonstrated in this figure, reaction rate follows second order kinetic and its values increases from 0.001/min to 0.041/min when initial dye concentration decreased from 600 to 50 mg/L in the solutions, respectively.

## Discussion

According to the obtained results, the most and the least important independent parameters were initial dye concentration and initial pH, respectively. Similar to our results, Aleboyeh *et al*. [22], Alinsafi *et al*. [21] and Arslan-Alaton *et al*. [23] study groups reported that initial pH was the least important parameter in comparison with the other variables. In addition, Durango-Usuga *et al*. [25] and Srivastava *et al*. [26] expressed that initial dye concentration is one of the most important factors in decolorization optimization respectively by Factorial and Taguchi designs, which is similar to our results.

Percentages of dyes removal in treatment by electrocoagulation process under optimized conditions through design of experiment methods (RSM, Taguchi and Factorial designs) are compared in Table 5. Present study shows 90.4% Acid Black 172 removal efficiency using electrocoagulation process through RSM at optimum point. As reported in Table 5, Alinsafi *et al*. [21] and Yildiz [27] achieved over 90% dyes removal efficiency at much higher reaction time and lower current density, respectively in comparison with the present study.

**Table 5:** Comparison of dye removal efficiency in treatment by electrocoagulation under optimal conditions through design of experiment methods

**Dye**	**Design**	Independent parameters^*^				**Dye removal efficiency(%)**	**Reference**
		**Current density (A**/**m**^**2**^**)**	**Reaction time (min)**	**Initial pH**	**Initial dye concentration (mg**/**L)**		
Acid Black 172	RSM	166.67	9.16	7	300	90.4	Present study
Acid Red 14	RSM	100	4	7	50	91.3	Aleboyeh *et al*. [22]
Reactive textile dyes	RSM	120	105	10	50	92	Alinsafi *et al*. [21]
Bomaplex Red CR-L	Taguchi	5	30	3	100	99.1	Yildiz [27]
Crystal Violet	Factorial	28	5	Natural	200	85	Durango-Usuga *et al*. [25]

* Independent parameters in present study under investigations optimal conditions.

Many Researchers have examined the impact of different parameters including initial pH, initial dye concentration, current density and reaction time on the dye removal efficiency in complex electrocoagulation process. Some study groups showed that the increase in current density and reaction time and the decrease in initial dye concentration improved the decolorization efficiency [6,19,22,28], which is similar to our results. However, optimum initial pH reported for different types of anionic dyes removal in electrocoagulation process was different. For example, optimum initial pH was reported 7, 5–9 and 4–6.5 by Aleboyeh *et al*. [22], Aoudj *et al*. [6] and Basiri Parsa *et al*. [20] study groups, respectively. Lower optimum initial pHs were also obtained by other researchers [26,27,29].

According to our knowledge, up to now there is no research available on treatment of Acid Black 172 in aqueous media by electrocoagulation procedure. Therefore, the observed data from our results have been compared with the other treatment methods of Acid Black 172. For instance, Du research group obtained 86% Acid Black 172 removal by Pseudomonas sp. DY1 at their optimum conditions through response surface methodology [30], which is close to our results.

## Conclusions

According to the results of this investigation, RSM is a powerful statistical optimization tool for Acid Black 172 removal using electrocoagulation process. The RSM results revealed that four selected parameters as well as some of their squares and interactions influenced the electrocoagulation performance. High R^2^ value of 94.48% through ANOVA, verified that the accuracy of the Minitab proposed polynomial model is acceptable. The optimum Acid Black 172 removal efficiency were found at initial pH of 7, initial dye concentration of 300 mg/l, applied current of 2 A and reaction time of 9.16 min. An experiment was performed in optimum conditions which confirmed that the model and experimental results are in close agreement (90.4% compared to 90% for the model).

## Competing interest

The authors also declare that they have no competing interests.

## Authors’ contributions

All authors read and approved the final manuscript.

## References

[B1] Rezaee A Ghaneian MT Khavanin A Hashemian SJ Moussavi GH Ghanizadeh GH Hajizadeh E Photochemical oxidation of reactive blue 19 dye (RB19) in textile wastewater by UV/K_2_S_2_O_8_ process Iran J Environ Health Sci Eng 2008 5 2 95 100

[B2] Sadri Moghaddam S Alavi Moghaddam MR Arami M Decolorization of an acidic dye from synthetic wastewater by sludge of water treatment plant Iran J Environ Health Sci Eng 2010 7 5 437 442

[B3] Ehrampoush MH Ghanizadeh GH Ghaneian MT Equilibrium and kinetics study of reactive Red 123 dye removal from aqueous solution by adsorption on eggshell Iran J Environ Health Sci Eng 2011 8 2 101 108

[B4] Mohammadian Fazli M Mesdaghinia AR Naddafi K Nasseri S Yunesian M Mazaheri Assadi M Rezaie S Hamzehei H Optimization of reactive blue 19 decolorization by ganoderma sp. using response surface methodology Iran J Environ Health Sci En 2010 7 1 35 42

[B5] Hasani Zonoozi M Alavi Moghadam MR Arami M Removal of acid red 398 dye from aqueous solutions by coagulation/flocculation pocess J Environ Eng & Manage 2008 7 6 695 699

[B6] Aoudj S Khelifa A Drouiche N Hecini M Hamitouche H Electrocoagulation process applied to wastewater containing dyes from textile industry Chem Eng Process 2010 49 11 1176 1182 10.1016/j.cep.2010.08.019

[B7] Yang Y Wang G Wang B Li Z Jia X Zhou Q Zhao Y Biosorption of acid black 172 and Congo Red from aqueous solution by nonviable penicillium YW 01: kinetic study, equilibrium isotherm and artificial neural network modeling, *bioresour* Technol 2011 102 2 828 834 10.1016/j.biortech.2010.08.125 20869234

[B8] Malakootian M Yousefi N The efficiency of electrocoagulation process using aluminum electrodes in removal of hardness from water Iran J Environ Health Sci Eng 2009 6 2 131 136

[B9] Murugan AA Ramamurthy T Subramanian B Kannan CS Ganesan M Electrocoagulation of textile effluent: RSM and ANN modeling Int J Chem React Eng 2009 7 10.2202/1542-6580.1942

[B10] Bhatti MS Kapoor D Kalia RK Reddy AS Thukral AK RSM and ANN modeling for electrocoagulation of copper from simulated wastewater: Multi objective optimization using genetic algorithm approach Desalination 2011 274 1–3 74 80

[B11] Bazrafshan E Mahvi AH Nasseri S Shaieghi M Performance evaluation of electrocoagulation process for diazinon removal from aqueous environments by using iron electrodes Iran J Environ Health Sci Eng 2007 4 2 127 132

[B12] Daneshvar N Khataee AR Amani Ghadim AR Rasoulifard MH Decolorization of C.I. Acid Yellow 23 solution by electrocoagulation process: Investigation of operational parameters and evaluation of specific electrical energy consumption (SEEC) J Hazard Mater 2007 148 3 566 10.1016/j.jhazmat.2007.03.028 17428605

[B13] Behbahani M Alavi Moghaddam MR Arami M Techno-economical evaluation of fluoride removal by electrocoagulation process: Optimization through response surface methodology Desalination 2011 271 1–3 209 218

[B14] Montgomery DC Design and analysis of experiments 2000 5 USA: John Wiley and Sons

[B15] Khuri AI (Ed): Response surface methodology and related topics 2006 Singapore: World Scientific

[B16] Ölmez T The optimization of Cr(VI) reduction and removal by electrocoagulation using response surface methodology J Hazard Mater 2009 162 2–3 1371 1378 18640776 10.1016/j.jhazmat.2008.06.017

[B17] Sadri Moghaddam S Alavi Moghaddam MR Arami M Response surface optimization of acid red 119 dye from simulated wastewater using Al based waterworks sludge and polyaluminium chloride as coagulant J Environ Manage 2011 92 4 1284 1291 10.1016/j.jenvman.2010.12.015 21216522

[B18] Balla W Essadki AH Gourich B Dassaa A Chenik H Azzi M Electrocoagulation/electroflotation of reactive, disperse and mixture dyes in an external-loop airlift reactor J Hazard Mater 2010 184 1–3 710 716 20870356 10.1016/j.jhazmat.2010.08.097

[B19] Mollah MYA Gomes JAG Das KK Cocke DL Electrochemical treatment of orange II dye solution—use of aluminum sacrificial electrodes and floc characterization J Hazard Mater 2010 174 1–3 851 858 19857925 10.1016/j.jhazmat.2009.09.131

[B20] Basiri Parsa J Rezaei Vahidian H Soleymani AR Abbasi M Removal of acid brown 14 in aqueous media by electrocoagulation: optimization parameters and minimizing of energy consumption Desalination 2011 278 1–3 295 302

[B21] Alinsafi A Khemis M Pons MN Leclerc JP Yaacoubi A Benhammou A Nejmeddine A Electro-coagulation of reactive textile dyes and textile wastewater Chem Eng Process 2005 44 4 461 470

[B22] Aleboyeh A Daneshvar N Kasiri MB Optimization of C.I. Acid Red 14 azo dye removal by electrocoagulation batch process with response surface methodology Chem Eng Process 2008 47 5 827 832 10.1016/j.cep.2007.01.033

[B23] Arslan-Alaton I Kobya M Akyol A Bayramoğlu M Electrocoagulation of azo dye production wastewater with iron electrodes: process evaluation by multi-response central composite design Color Technol 2009 125 4 234 241 10.1111/j.1478-4408.2009.00202.x

[B24] Nourouzi MM Chuah TG Choong TSY Optimisation of reactive dye removal by sequential electrocoagulation–flocculation method: comparing ANN and RSM prediction Water Sci Technol 2011 63 5 985 995 10.2166/wst.2011.280 21411950

[B25] Durango-Usuga P Guzmán-Duque F Mosteo R Vazquez MV Peñuela G Torres-Palma RA Experimental design approach applied to the elimination of crystal violet in water by electrocoagulation with Fe or Al electrodes J Hazard Mater 2010 179 1–3 120 126 20303653 10.1016/j.jhazmat.2010.02.067

[B26] Srivastava VC Patil D Srivastava KK Parameteric optimization of dye removal by electrocoagulation using Taguchi methodology Int J Chem React Eng 2011 9 10.1515/1542-6580.2299

[B27] Yildiz YS Optimization of bomaplex Red CR-L dye removal from aqueous solution by electrocoagulation using aluminum electrodes J Hazard Mater 2008 153 1–2 194 200 17875363 10.1016/j.jhazmat.2007.08.034

[B28] Sengil IA Ozacar M The decolorization of C.I. Reactive black 5 in aqueous solution by electrocoagulation using sacrificial iron electrodes J Hazard Mater 2009 161 2–3 1369 1376 18550279 10.1016/j.jhazmat.2008.04.100

[B29] MartÍnez-Huitle CA Brillas E Decontamination of wastewaters containing synthetic organic dyes by electrochemical methods: a general review Appl Catal, B Environ 2009 87 3–4 105 145

[B30] Du LN Yang YY Li G Wang S Jia XM Zhao YH Optimization of heavy metal-containing dye acid black 172 decolorization by pseudomonas sp. DY1 Using statistical designs Int Biodeter Biodeg 2010 64 7 566 573 10.1016/j.ibiod.2010.06.009

